# P02.140. Comparing the effect of ozonated olive oil to clotrimazole cream in the treatment of vulvovaginal candidiasis

**DOI:** 10.1186/1472-6882-12-S1-P196

**Published:** 2012-06-12

**Authors:** F Tara, Z Zand-Kargar, O Rajabi, F Berenji, H Azizi

**Affiliations:** 1 Mashhad University of Medical Sciences, Mashad, Iran

## Purpose

Vulvovaginal candidiasis is the most common infection of the vulvovagina which manifests with itching, burning sensation and leucorrhea. Conventional treatments are azoles to which tolerance has been reported, especially in immunosuppressed patients. New studies suggest antifungal effects of ozone, the allotropic form of oxygen. This study compared the effects of ozononated olive oil and clotrimazole in the treatment of vulvovaginal candidiasis.

## Methods

One hundred patients with confirmed vulvovaginal candidiasis were randomly classified to two groups and treated by ozonated olive oil or clotrimazole for 7 days. The study outcomes were changes in itching, burning, leucorrhea and culture before and after the treatment, which were evaluated by an interview and paraclinical examination. Statistical analysis was done by SPSS software, version 17. The significance level stood at 0.05.

## Results

Ozone and clotrimazole both reduced the symptoms significantly and led to negative specimen cultures (p<0.05). There was no significant difference between the two groups in their effect on itching, leucorrhea and culture (p>0.05). However, ozone decreased burning sensation significantly better than clotrimazole (p<0.05).

## Conclusion

Considering the potential efficacy of ozonated olive oil for the improvement of clinical and paraclinical aspects of patients with vulvovaginal candidiasis, it could be suggested as an effective topical treatment for these patients.

